# Pretreatment Thrombocytosis as a Prognostic Factor in Metastatic Breast Cancer

**DOI:** 10.1155/2013/289563

**Published:** 2013-06-24

**Authors:** Athina Stravodimou, Ioannis A. Voutsadakis

**Affiliations:** Department of Medical Oncology, University Hospital of Lausanne, 1011 Lausanne, Switzerland

## Abstract

*Background*. An elevated platelet count is often associated with malignancies, and it has been confirmed as an adverse prognostic factor in various cancers including early stage breast cancer. We sought to determine if thrombocytosis is also a prognostic factor in metastatic breast cancer. *Patients and Methods*. The records of 165 metastatic breast cancer patients with complete follow-up that had thrombocytosis or normal platelet counts were reviewed. Kaplan-Meier curves were constructed, and the survivals of the two groups were compared using the LogRank test. A Cox regression analysis was used to determine if thrombocytosis is an independent factor for overall and progression free survival. *Results*. There was a statistically significant difference in overall and progression free survival favoring the normal platelets group (LogRank test *P* = 0.038 and 0.008, resp.). Thrombocytosis remained a significant adverse prognostic factor in multivariate analysis. Other independent prognostic factors for overall survival included age, ER/PR status, and grade. *Conclusion*. Thrombocytosis represents an independent adverse prognostic factor in patients with metastatic breast cancer. Thus metastatic breast cancer joins a range of cancers in which this easily measurable value can be used for clinical prognostication. Further use as a predictive value for specific treatments has a rationale and deserves to be investigated.

## 1. Introduction 

Platelets are important cellular particles for hemostasis and vascular integrity. They are produced from bone marrow precursor cells, megakaryocytes. Abnormalities in their normal circulating number either in the form of thrombocytosis or thrombocytopenia are associated with many pathologic conditions [1]. Cytokines that stimulate thrombopoiesis are often elevated in cancer, and as a result, various cancers have been associated with thrombocytosis. In addition thrombocytosis has been found to be an adverse prognostic factor in many types of common cancers.

 Breast cancer is the most common female malignancy. When localized, it is treated with surgery and often with adjuvant therapies to decrease the risk of local or systemic recurrences [2]. Both adjuvant therapies and therapies in the metastatic disease setting are guided by biologic characteristics such as hormone receptors and growth factor Her-2 expression. These characteristics possess also prognostic information, but additional markers are needed to further promote prognostication of breast cancer. In early stage breast cancer patients undergoing chemotherapy in five clinical trials of the Austrian Breast and Colorectal Cancer Study Group, pretreatment thrombocytosis was an independent prognostic factor for overall survival and breast cancer-specific survival [3]. We investigated if pretreatment high thrombocyte counts provide prognostic information in patients with metastatic breast cancer.

## 2. Patients and Methods

 Case records of women with breast cancer treated in the Medical Oncology Department of the University Hospital of Lausanne over the last 12 years were retrospectively reviewed. Further information was collected from charts of patients with metastatic disease at diagnosis or at any later time of their disease history and with complete follow-up. Follow-up was considered complete if a patient was followed till her death or was seen within the last 6 months from data collection. Data on patients' age, biologic characteristics of tumors, time from original diagnosis to metastatic disease, and site(s) of metastases were recorded. Platelet number on diagnosis of metastatic breast cancer (before the start of any therapy for metastatic disease) was evaluated in 179 patients for whom there were complete follow-up data. Overall survival (OS) was defined as the interval from the date of diagnosis of metastatic disease to death. Progression free survival (PFS) was defined as the interval from the date of diagnosis of metastatic disease to disease progression or death, whichever happened first. Her-2/Neu positivity was defined as 3+ by immunohistochemistry (IHC) or FISH amplification according to standard criteria [4]. For the purposes of the current study Her-2 was considered negative if IHC was 2+ and FISH had not been performed. In the normal platelet count group patients with platelet counts of 150 to 350 × 10^9^/L were included. In the thrombocytosis group patients with platelet counts of >350 × 10^9^/L were included. Survival plots of patients with normal platelet counts and thrombocytosis were constructed using the Kaplan-Meier method and were compared using the LogRank test [5]. The *χ* ^2^ test was used to evaluate differences in clinical and biologic characteristics in the two groups [6]. A Cox regression proportional hazard multivariate analysis was performed to identify statistically significant factors associated with overall and progression free survival. All *P* values were considered to be significant at the level of *P* < 0.05. Statistical calculations were performed with online tools available from the Technical University of Denmark (http://www.iscc-serv2.imm.dtu.dk/) and a noncommercial site (http://www.statpages.org/).

## 3. Results 

 Among 215 patients with metastatic breast cancer followed in our department during the period of the last 12 years, 179 patients had complete follow-up. Fourteen patients had thrombocytopenia at diagnosis and were excluded from further analysis. From the remaining 165 patients, 135 (81.8%) had normal platelet counts at diagnosis of metastatic disease, and 30 (18.2%) had thrombocytosis (Table 1). The median platelet count of the whole group of patients was 261 × 10^9^/L (range 154–694). The median platelet count in the normal platelet group was 241 × 10^9^/L (range 154–349) and in the thrombocytosis group was 407 × 10^9^/L (range 354–694). The median age of the whole group was 62 years old (range 31–92). 110 patients had died, and 55 patients were alive at last follow-up. The median follow-up of patients alive was 25 months and the mean 30.14 months [95% confidence interval (CI) 23.55–36.74]. The median age of the patients with normal counts was 62 years old (range 31–92) and of those with thrombocytosis was 66 years old (range 44–85). In the normal platelets group 92 patients had died, and 43 patients were alive with a median follow-up of 33 months (mean 33.88 months, 95% CI 25.95–41.82). In the thrombocytosis group 18 patients had died and 12 patients were alive with a median follow-up of 12.5 months (mean 16.75 months, 95% CI 9.33–24.17). Other baseline characteristics of patients and their disease are given in Table 1. There were no statistically significant differences in the proportion of patients in the two groups regarding the hormone receptors status, the Her-2 status, the grade of the tumors, whether metastatic disease was confined to bone or had also spread to other sites, and the therapy patients had received as first line metastatic treatment. For patients that had initially been diagnosed with local disease (*n* = 98), there was no difference between the normal platelet and thrombocytosis group in the percentage of patients who received no adjuvant treatment or only adjuvant hormonotherapy and those that had chemotherapy included in their adjuvant treatment (*P* = 0.87). More patients in the thrombocytosis group had metastatic disease at diagnosis (70% versus 34.1% in the normal platelets group, *P* = 0.0003).

 The median overall survival of patients that had died in the whole group (110 of 165 patients) was 24 months, and the mean was 28.2 months (95% CI 23.7–32.7). The median overall survival (OS) of patients that had died in the group with normal platelet counts was 26 months (range 1–115 months) and the mean was 30.08 months (95% CI 24.97–35.19) while the median OS of patients with thrombocytosis was 12.5 months (range 2–54 months) and the mean was 18.6 months (95% CI 10.95–26.27). OS was 50% (95% CI 41.6–58.3%) and 26% (95% CI 14–44.6%) at 3 years in the normal and thrombocytosis group, respectively. There were no long term survivors at 5 year in the group with thrombocytosis while in the normal platelets group 5-years survival was 22% (95% CI 15.35–29.2%).

**Figure 1:**
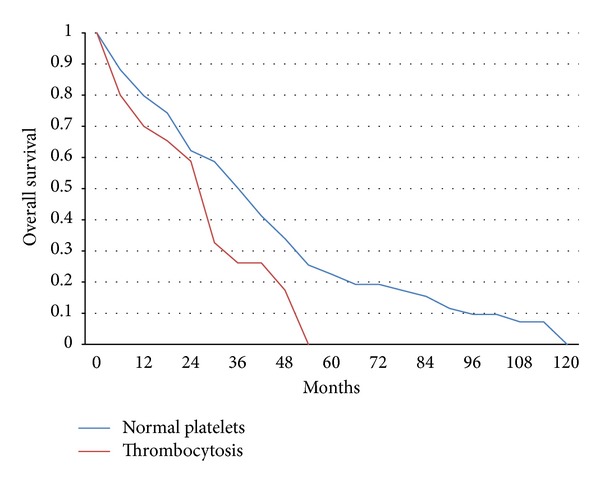
Kaplan-Meier overall survival curves in months from the diagnosis of metastatic disease of patients with normal platelet counts (150–350 × 10^9^/L) versus patients with thrombocytosis. LogRank test *P* = 0.038.

 There was a statistically significant difference of the overall survival between the two groups (LogRank test *P* = 0.038) (Figure 1). In the multivariate Cox regression analysis, increasing age, higher grade, ER/PR negativity, and thrombocytosis were statistically significantly associated with reduced overall survival while Her-2 status, the presence of metastases at diagnoses, the location of metastatic disease, and the administration and type of first line metastatic treatment were not (Table 2).

**Figure 2:**
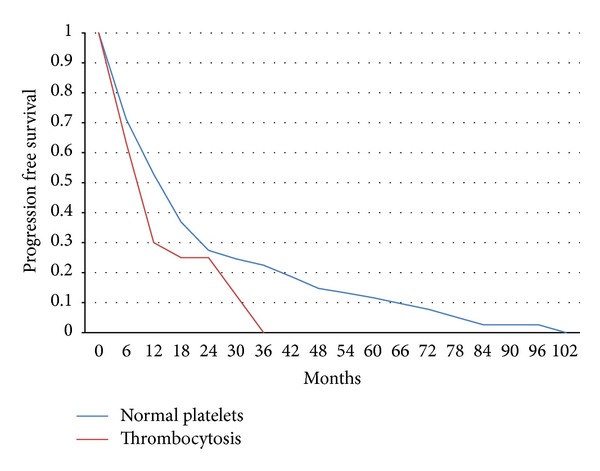
Kaplan-Meier progression free survival curves in months from the diagnosis of metastatic disease of patients with normal platelet counts (150–350 × 10^9^/L) versus patients with thrombocytosis. LogRank test *P* = 0.008.

 There was also a statistically significant difference of the progression free survival between the two groups (LogRank test *P* = 0.008) (Figure 2). In this instance the multivariate Cox regression analysis showed that higher grade, ER/PR negativity, the presence of metastases at diagnoses, and thrombocytosis were statistically and significantly associated with reduced progression free survival while age, Her-2 status, the location of metastatic disease, and the administration and type of first line metastatic treatment were not (Table 3).

## 4. Discussion

 An elevated platelet count may have various causes and is either primary due to essential thrombocytosis or other myeloproliferative disorders or secondary to infection, trauma or surgery, iron deficiency, or malignancy. When other conditions are excluded, about 40% of patients with thrombocytosis of more than 400 × 10^9^/L have been found to harbor an occult cancer [7]. In this retrospective study we have shown for the first time that thrombocytosis is associated with decreased OS and PFS in patients with metastatic breast cancer. In our cohort of 165 patients there were no significant differences between the two groups with normal platelets and thrombocytosis in the age of patients, biologic characteristics of their cancer such as ER/PR and Her-2 expression and grade, and no significant difference on whether they had bone only or other sites of metastases. Patients with thrombocytosis were more likely to have metastases at diagnosis with breast cancer while the normal platelet group was more likely to have developed metastatic disease at a later time in the course of their disease (*P* = 0.0003). This may be due to a more aggressive biology of cases with thrombocytosis that favors earlier metastases development. In the multivariate analysis, increasing age, hormonal receptors negativity, and higher grade were associated together with thrombocytosis with decreased overall survival. Her-2 was not an independent predictor of overall or progression free survival possibly due to the fact that, for a significant minority of patients (23 out of 165), Her-2 status was unknown or ambiguous (meaning that it was 2+ by IHC, and no FISH was performed) or due to treatment with anti-Her-2 targeted therapies which improve the outcome of these patients [8, 9]. Bone only disease also was not a significant prognostic factor in the multivariate analysis possibly due to its association with other parameters and notably hormone receptor status.

 Other investigators have previously detected an association of thrombocytosis with worse outcome in patients with early breast cancer [3]. This retrospective study is the most extended series addressing the subject in any type of cancer and included 4,300 patients that had been treated in five randomized trials of the Austrian Breast and Colorectal Cancer Study Group. In this report thrombocytosis was defined as platelets more than 400 × 10^9^/L and was present in 3.7% of patients. The estimated overall survival of patients with thrombocytosis was 71 months versus 99.5 months in patients without thrombocytosis. Thrombocytosis was an independent prognostic factor for overall survival and breast cancer related survival but not for disease-free survival [3].

 Thrombocytosis appears to be a universal marker of adverse outcomes in cancer. Its association with worse oncologic outcomes has been also reported in esophageal squamous cell carcinoma [10], gastric adenocarcinoma [11], renal cell carcinoma [12], ovarian carcinoma [13], and other cancers [14, 15]. In patients with esophageal squamous cell carcinoma, thrombocytosis, defined as platelets more than 293 × 10^9^/L which was the mean plus 1 standard deviation of a healthy control group, was present in 21% of patients and was a significant independent prognostic factor [10]. This association was statistically significant for patients with stages III and IV but not for stages I and II disease. In gastric adenocarcinoma patients that had undergone gastrectomy with negative margins, thrombocytosis (defined as platelets more than 400 × 10^9^/L in this study) was associated with worse survival and was a strong predictor specifically of hematogenous metastasis but not of locoregional recurrence or peritoneal seeding [11]. In another series of gastric cancer patients, thrombocytosis was associated with worse 1-year and 3-year survival and was positively correlated with depth of tumor invasion [16]. A third example of tumor where thrombocytosis has prognostic significance is renal cell carcinoma. Metastatic renal carcinoma patients with thrombocytosis (defined as platelets more than 400 × 10^9^/L at least in one occasion during their disease) had a mean survival of 92 months compared with 151 months in patients with normal platelets [12]. This difference was significant in multivariate Cox analysis.

 Thrombocytosis (defined as platelets more than 450 × 10^9^/L) was associated with more advanced stage and higher preoperative Ca-125 in ovarian carcinoma [13]. In addition the median overall survival in patients with thrombocytosis was 2.62 years while in the group with normal platelets it was 4.65 years (*P* < 0.001). In another study in ovarian cancer, thrombocytosis (defined as platelets more than 400 × 10^9^/L) was also associated with advanced stage and grade and reduced overall survival [17].

 Thrombocytosis was significantly correlated with plasma levels of IL-6 in patients with ovarian carcinoma [13]. In mouse models bearing human ovarian cancer, human IL-6 was found to stimulate hepatocytes via the IL-6 receptor to produce thrombopoietin. Thus a model was proposed according to which ovarian cancer tumor cells produce IL-6 which stimulates the liver to produce thrombopoietin, finally resulting in increased thrombopoiesis through stimulation of megakaryocyte progenitors in the bone marrow [13]. In other cancers IL-6 may also play a similar role in producing thrombocytosis. In renal carcinoma the majority of cases examined were positive for IL-6 by immunohistochemistry [18]. Serum levels of IL-6 were elevated in prostate cancer patients with more advanced stage disease and correlated with decreased disease-specific survival [19]. In breast cancer serum IL-6 levels were significantly higher than in a control group of healthy women [20]. Thus IL-6 produced from tumor cells may be a pathophysiologic trigger of tumor-induced thrombocytosis across different cancer types in a manner similar to that proposed for ovarian cancer [13].

The mechanistic basis of platelets contribution to carcinogenesis is a subject of study [21]. Circulating tumor cells may use platelets as a shield to protect themselves from the attack of the immune system and as an intermediary helping them to attach to endothelial cells at the destination sites of metastases. In addition heteroaggregates constituted of platelets and tumor cells may embolize in the microcirculation and aid in the process of extravasation of tumor cells in metastatic sites. Platelets have also roles in carcinogenesis directly related to their normal function in promotion of vascular integrity [22]. Newly formed tumor vasculature is particularly prone to dysfunction, and platelets have been shown to be indispensable for preventing hemorrhage in tumor beds [23]. Platelets are carriers in their granules of a plethora of bioactive molecules and growth factors. These include Vascular Endothelial Growth Factor (VEGF), Epidermal Growth Factor (EGF), Platelet-Derived Growth Factor (PDGF), Hepatocyte Growth Factor (HGF), Insulin-like Growth Factor (IGF), Transforming Growth Factor *β* (TGF*β*), Interleukin 1*β* (IL-1*β*), IL-8, CXC motif containing ligand 12 (CXCL12), Sphingosine-1-phosphate (S1P), and lysophosphatidic acid among others [24, 25]. Each of these factors may actively contribute to metastatic tumor progression. Platelet-derived TGF*β*, for example, promotes an EMT (epithelial to mesenchymal transition) program in tumor cells through Smad and NF-*κ*B signaling in these cells [26]. EMT is a program that endows epithelial cells with a mesenchymal phenotype that promotes mobility and metastasis while protecting them from anoikis (apoptosis due to lack of adhesion) [27]. Platelets from patients with cancer have a higher VEGF level than platelets from individuals without cancer [28]. In contrast circulating VEGF is not elevated in cancer patients except in renal cell carcinoma if care is taken to avoid artificial platelet activation during venipuncture [28]. As a result platelet counts may better reflect VEGF concentrations in the tumor and metastases sites environment where they are activated and contribute to tumor angiogenesis. 

In conclusion, this retrospective analysis of a series of metastatic breast cancer patients shows that thrombocytosis (defined in this paper as platelets more than 350 × 10^9^/L) at the time of diagnosis of metastatic disease has prognostic value regarding overall and progression free survival. The presence of thrombocytosis is independent of biologic characteristics that help classify breast cancer to subgroups relevant for treatment such as ER/PR and Her-2 expression and thus has value beyond these characteristics. Further study is needed in more extensive series to confirm these results and especially to test whether thrombocytosis can serve as a predictive marker of specific treatments. In this respect and in view of the above discussion it would be of particular interest to test thrombocytosis as a predictive marker of anti-VEGF therapies. Indeed a recent study in metastatic renal cell carcinoma has shown that patients with thrombocytosis had a higher risk to present a primary refractoriness to anti-VEGF treatments (odds ratio = 1.7, *P* = 0.0068) than patients with normal platelets [29]. It remains to be seen if thrombocytosis could be a predictive factor for anti-VEGF therapies in other cancers and in breast cancer in particular.

## Figures and Tables

**Table 1:** Characteristics and outcome of patients in the series. Column “All patients” includes the whole series of patients with normal platelets or thrombocytosis. *χ* ^2^ test between the group with normal platelet count and the group with thrombocytosis is shown. Wherever there are more than two categories, the grouping of the comparison is mentioned in the same column. Her-2 was defined as positive if 3+ by IHC or amplified by FISH and negative if 1+ by IHC or 2+ by IHC and FISH not amplified or not performed.

	All patients	normal	thrombocytosis	*χ* ^2^
Number (%)	165 (100)	135 (81.8)	30 (18.2)	
Age at diagnosis of metastatic disease				
Median (range)	62 (31–92)	62 (31–92)	66 (44–85)	
>65 years	70 (42.4)	54 (40)	16 (53.3)	*P* = 0.17
≤65 years	95 (57.6)	81 (60)	14 (46.7)	
ER/PR				Positive versus negative *P* = 0.69
Positive (either or both)	126 (76.4)	102 (75.5)	24 (80)	
Negative (both)	37 (22.4)	31 (23)	6 (20)	
Unknown	2 (1.2)	2 (1.5)	0	
Her-2				Positive versus negative *P* = 0.35
Positive	35 (21.2)	27 (20)	8 (26.7)	
Negative	107 (64.9)	90 (66.7)	17 (56.7)	
Unknown or ambiguous	23 (13.9)	18 (13.3)	5 (16.6)	
Grade				III versus I and II *P* = 0.42
I	10 (6.1)	8 (6)	2 (6.7)	
II	66 (40)	55 (40.7)	11 (36.6)	
III	67 (40.6)	52 (38.5)	15 (50)	
Unknown	22 (13.3)	20 (14.8)	2 (6.7)	
Metastatic at diagnosis				
Yes	67 (40.6)	46 (34.1)	21 (70)	*P* = 0.0003
No	98 (59.4)	89 (65.9)	9 (30)	
Sites of metastases				Bone only versus other *P* = 0.81
Bone	41 (24.9)	34 (25.2)	7 (23.3)	
Soft tissue	21 (12.7)	20 (14.8)	1 (3.3)	
Parenchymal	38 (23)	34 (25.2)	4 (13.4)	
Multiple sites	65 (39.4)	47 (34.8)	18 (60)	
1st metastatic treatment				
None or hormonotherapy	74 (44.8)	63 (46.7)	11 (36.7)	*P* = 0.32
Chemotherapy	91 (55.2)	72 (53.3)	19 (63.3)	
Adjuvant treatment				
None or hormonotherapy	52 (53.1)	47 (52.8)	5 (55.6)	*P* = 0.87
Chemotherapy	46 (46.9)	42 (47.2)	4 (44.4)	

**Table 2:** Multivariate Cox regression analysis of parameters possibly related to overall survival of metastatic breast cancer patients.

Variable	Hazard ratio	95% confidence interval		*P* value
		Lower limit	Upper limit	
Thrombocytosis	1.75	1.01	3.02	0.043
Age	1.02	1.011	1.044	0.0009
ER/PR status	0.50	0.31	0.78	0.0028
Her-2 status	0.93	0.57	1.51	0.77
Grade	1.48	0.98	2.21	0.05
Metastatic at diagnosis	0.82	0.54	1.25	0.36
Site of metastasis (bone only versus other)	0.79	0.48	1.29	0.34
1st metastatic treatment (none or hormonotherapy versus chemotherapy)	1.47	0.92	2.33	0.10

**Table 3:** Multivariate Cox regression analysis of parameters possibly related to progression free survival of metastatic breast cancer patients.

Variable	Hazard ratio	95% confidence interval		*P* value
		Lower limit	Upper limit	
Thrombocytosis	2.00	1.26	3.17	0.0031
Age	1.00	0.9975	1.023	0.21
ER/PR status	0.59	0.38	0.91	0.018
Her-2 status	1.027	0.65	1.61	0.90
Grade	1.54	1.06	2.22	0.021
Metastatic at diagnosis	0.61	0.42	0.89	0.012
Site of metastasis (bone only versus other)	0.82	0.52	1.30	0.40
1st metastatic treatment (none or hormonotherapy versus chemotherapy)	1.37	0.87	2.16	0.17
